# Can Neuron Specific Enolase Be a Diagnostic Biomarker for Neuronal Injury in COVID-19?

**DOI:** 10.7759/cureus.11033

**Published:** 2020-10-19

**Authors:** Latha Ganti, Enrique Serrano, Hale Z Toklu

**Affiliations:** 1 Emergency Medicine, University of Central Florida College of Medicine, Orlando, USA; 2 Neurology, University of Central Florida College of Medicine, Orlando, USA; 3 Clinical Sciences, University of Central Florida College of Medicine, Orlando, USA; 4 Graduate Medical Education, Hospital Corporation of America North Florida Division, Gainesville, USA

**Keywords:** neuron specific enolase, nse, cerebrospinal fluid, csf, covid-19, sars-cov2

## Abstract

Neuron specific enolase (NSE) is a biomarker for neuronal injury. However, increased levels in cerebrospinal fluid (CSF) and serum is associated with the clinical outcome in patients with head injury, ischemic stroke, intracerebral hemorrhage, cardiac arrest, anoxic encephalopathy, encephalitis, brain metastasis, and status epilepticus.

Recently, the severe acute respiratory syndrome coronavirus 2 (SARS-CoV-2) infection, which started in China, rapidly evolved into the coronavirus disease 2019 (COVID-19) pandemic. Patients with COVID-19 have a wide range of symptoms varying from mild upper respiratory symptoms to severe illness requiring mechanical ventilation. While coronaviruses primarily target the human respiratory system, neurological symptoms are also observed in some patients. These include symptoms such as loss of taste and olfaction and diseases like cerebrovascular disorders including ischemic stroke and hemorrhages, encephalopathies, Guillain‐Barré syndrome and acute disseminated encephalomyelitis.

Here we report an observation from a patient whose NSE levels increased approximately four-fold in CSF. This finding was accompanied by increased white blood cell count and elevated protein in CSF indicating neuroinflammation. Thus, we suggest that NSE may be used as a CSF biomarker in COVID-19 patients with encephalopathy.

## Introduction

Neuron-specific enolase (NSE) is a glycolytic enzyme, which is expressed abundantly in the neurons and neuroendocrine cells. It was first used as a tumor marker for small-cell lung cancer, neuroblastoma and other malignancies of neuroendocrine origin, and later it was introduced as a marker for brain damage [1], because it is found that the NSE levels increase in serum and cerebrospinal fluid (CSF) following acute neurotrauma as an indicator for neuronal injury and synaptic dysfunction [2]. NSE is located in the cytoplasm of the neurons. The disruption of the blood-brain barrier integrity and damage to the neuronal tissue causes the release of NSE into the CSF and then the blood [3]. Therefore the level of NSE correlates with damage [4]. NSE levels was shown to be associated with the clinical outcome in patients with serious clinical manifestations such as head injury, ischemic stroke, intracerebral hemorrhage, cardiac arrest, anoxic encephalopathy, encephalitis, brain metastasis, and status epilepticus [5]. 

Recently, the severe acute respiratory syndrome coronavirus 2 (SARS-CoV-2) infection, which started in China, rapidly evolved into the coronavirus disease 2019 (COVID-19) pandemic. Patients with COVID-19 have a wide range of symptoms varying from mild upper respiratory symptoms to severe illness requiring mechanical ventilation. Neurological symptoms are also observed in some patients. Previous evidence suggested that studies on the samples from patients with severe acute respiratory syndrome (SARS) have demonstrated the presence of SARS coronavirus (SARS-CoV) particles in the brain, where they were located almost exclusively in the neurons. Thus, the respiratory failure could be partially due to the neuroinvasion of the brainstem by the virus [6,7].

## Case presentation

A 77-year-old white male patient admitted to the hospital with headache, nausea, respiratory distress, and pneumonia. Patient was diagnosed with COVID-19, which was confirmed with the polymerase chain reaction (PCR) from the nasopharyngeal swab. The patient had a history of atherosclerotic heart disease of native coronary artery without angina pectoris, fatty liver, and gout.

The patient had acute respiratory distress and required ventilator support on day two which continued for the next 17 days. He died afterward on day 20 of the hospitalization. The patient developed hypernatremia on day eight, which was treatment-resistant and continued until day 19. His sodium levels were >151 mEq/L from day 11 to the end. The patient also developed acute renal failure and hepatic failure due to sepsis. (Day 16-17: alanine aminotransferase (ALT): 207; aspartate aminotransferase (AST): 186; Creatinine: 1.58; white blood cells (WBCs): 12,500). The patient was evaluated for meningoencephalitis and electroencephalogram (EEG) monitoring was performed for seizures.

The patient demographics, admission vitals, laboratory findings in serum and cerebrospinal fluid (CSF) analysis, and clinical outcome are summarized in Table 1. 

**Table 1:** The demographic characteristic, vitals, laboratory findings in serum and cerebrospinal fluid (CSF) analysis and clinical outcome

		Result	Normal Range/ Remarks
Demographics	Age	77	
	Sex	Male	
	Race	White	
Vitals (admission)	Oxygen saturation SPO2 (%)	91	95-100 %
	Temperature	97.5	97-99 F
	Pulse	71	60-80 beats/min
	Blood pressure	139/68	120/ 80 mmHg
Serum (Admission)	White blood cell count	2.99	3.80-10.80
	Platelet	81	140-400
	Hemoglobin	15.7	13.7-17.7 g/dl
	D-dimer (day 3)	1.62	0.15-0.50 mg/L FEU
	Troponin I	0.018	0.000-0.045 ng/ml
	Alanine aminotransferase (ALT)	32	16-63 (U/L)
	Aspartate aminotransferase (AST)	54	15-37 U/L)
	Creatinine	0.89	0.60-1.30 mg/ dL
	Sodium (admission)	139	136-145 (mEq/L)
	Sodium (day 16)	156	136-145 (mEq/L)
CSF (day 17)	Appearance	Clear	Clear
	Color	Colorless	Colorless
	Glucose (mg/dl)	107	40-70 mg/dL
	Lymphocyte count	75	40-80 %
	Monocytes and macrophages	25	15-45 %
	Polymorponuclear cell count	0	<6 %
	Nucleated cell count	10	0-5 /mcL
	Erythrocyte count	12	0-10 /mcl
	Total Protein (mg/dl)	51.4	15-45mg/dl
	Immunoglobulin G	3.95	0.0-3. 4 mg/dL
	Neuron specific enolase (NSE)	33	<8.9 ng/ ml
	VDRL (venereal disease research laboratory)	Negative	Negative
	Herpes simplex virus (HSV) HSV1 HSV2	Negative	Negative
Clinical outcome	Neurological symptoms	Present Nausea, Seizures, urinary incontinence	
	Other diagnosis	Sepsis, Acute respiratory distress syndrome, Acute kidney failure Acute liver failure	
	Disease severity	Critically ill	
	Ventilator need	Yes	
	Ventilator time	17 days	
	Length of stay in hospital	20	
	In-hospital mortality	Expired	

**Figure 1:**
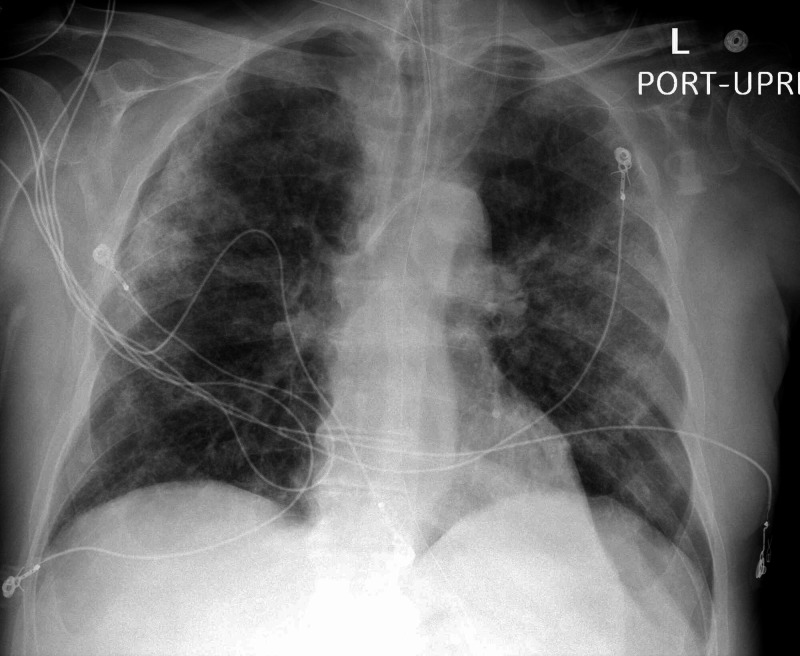
Chest x-ray of the patient indicating COVID-19 pneumonia

The patient's chest X-ray which indicates pneumonia and ground-glass opacities in the lung is shown in Figure 1. 

## Discussion

Unlike bacterial infections, viral infections are less likely to cause remarkable changes in CSF glucose, cell count, or protein [8]. In this COVID-19 case, CSF findings such as increase in total protein, total cell count, and immunoglobulin G (IgG) indicated the presence of inflammation in the central nervous system. The marked increase (approximately four-fold) in NSE levels in CSF further supported the inflammatory invasion and neuronal injury. Additionally, this patient had persistent hypernatremia, which continued for over 10 days, which may have contributed to the encephalopathy and osmotic demyelination.

## Conclusions

NSE is an old marker, which is sensitive but poorly specific. Nevertheless, the findings are in tune with prior literature as NSE is a marker of neuronal damage. Based on this single observation, we suggest that NSE can be a candidate for a diagnostic/prognostic biomarker for neuroinflammation in COVID-19, especially for patients who have neurological symptoms. However, a study with a larger cohort is needed to evaluate the clinical significance. 

## References

[1] Isgrò MA, Bottoni P, Scatena R (2015). Neuron-specific enolase as a biomarker: biochemical and clinical aspects. Adv Exp Med Biol.

[2] Haque A, Polcyn R, Matzelle D, Banik NL (2018). New insights into the role of neuron-specific enolase in neuro-inflammation, neurodegeneration, and neuroprotection. Brain Sci.

[3] Lamers KJ, Vos P, Verbeek MM, Rosmalen F, van Geel WJ, van Engelen BG (2003). Protein S-100B, neuron-specific enolase (NSE), myelin basic protein (MBP) and glial fibrillary acidic protein (GFAP) in cerebrospinal fluid (CSF) and blood of neurological patients. Brain Res Bull.

[4] Marchi N, Rasmussen P, Kapural M (2003). Peripheral markers of brain damage and blood-brain barrier dysfunction. Restor Neurol Neurosci.

[5] Lima JE, Takayanagui OM, Garcia LV, Leite JP (2004). Use of neuron-specific enolase for assessing the severity and outcome in patients with neurological disorders. Braz J Med Biol Res.

[6] Baig AM, Khaleeq A, Ali U, Syeda H (2020). Evidence of the COVID-19 virus targeting the CNS: tissue distribution, host-virus interaction, and proposed neurotropic mechanisms. ACS Chem Neurosci.

[7] Li YC, Bai WZ, Hashikawa T (2020). The neuroinvasive potential of SARS-CoV2 may play a role in the respiratory failure of COVID-19 patients. J Med Virol.

[8] Hrishi AP, Sethuraman M (2019). Cerebrospinal fluid (CSF) analysis and interpretation in neurocritical care for acute neurological conditions. Indian J Crit Care Med.

